# Premature ovarian insufficiency: Updated concepts in diagnosis and hormonal treatment strategies

**DOI:** 10.1016/j.clinsp.2025.100745

**Published:** 2025-08-20

**Authors:** Gabriela Pravatta-Rezende, Cristina Laguna Benetti-Pinto, Daniela Angerame Yela, Ana Carolina Japur de Sá Rosa e Silva, Andrea Prestes Nácul, Fernando Marcos dos Reis, Edmund Chada Baracat, José Maria Soares Junior

**Affiliations:** a Department of Obstetrics and Gynecology, Universidade de Campinas, Campinas, SP, Brazil; b Member of the National Specialized Commission on Endocrine Gynecology of the Brazilian Federation of Gynecology and Obstetrics (FEBRASGO), São Paulo, SP, Brazil; c Department of Gynecology and Obstetrics, Universidade de São Paulo, São Paulo, SP, Brazil; d Human Reproduction Unit, Hospital Fêmina, Grupo Hospitalar Conceição, Porto Alegre, RS, Brazil; e Department of Gynecology and Obstetrics, Faculdade de Medicina da Universidade Federal de Minas Gerais (UFMG), Belo Horizonte, MG, Brazil

**Keywords:** Premature ovarian insufficiency, Amenorrhea, Hormone therapy, Hypoestrogenism, Estradiol

## Abstract

**Objective:**

To provide an updated overview of Premature Ovarian Insufficiency (POI), focusing on the current evidence regarding diagnosis, hormonal treatment, and clinical management.

**Methods:**

A narrative review was conducted using data from the FEBRASGO protocol and updated international guidelines, complemented by recent publications from PubMed and Scopus.

**Results:**

POI affects up to 3.5 % of women under 40-years of age and is associated with significant physical and psychological repercussions due to early hypoestrogenism. The diagnosis is established based on persistent amenorrhea and elevated FSH levels, and etiological diagnosis should be encouraged. Hormone Therapy (HT) is the cornerstone of management and must be continued at least until the age of natural menopause. New evidence emphasizes the use of l17β-estradiol (preferably non-oral route) combined with cyclic or continuous progestogens. Fertility preservation and sexual health should be addressed early in management.

**Conclusion:**

Hormonal replacement in POI is essential to prevent long-term complications and improve quality of life. Guidelines for healthy lifestyle habits should be reinforced and clinical decisions should be individualized, guided by age, symptom profile, and reproductive plans.

## Highlights

• Diagnosis and etiology of POI.
• Hormone therapy as an essential treatment.
• Fertility preservation strategies.
• Long-term monitoring and multidisciplinary care.
• Androgen therapy for sexual dysfunction.

## Introduction

Premature Ovarian Insufficiency (POI) is a condition resulting from the loss of ovarian activity before the age of 40, due to a low follicular pool, accelerated follicular atresia, or impaired folliculogenesis. The clinical symptoms reflect a state of hypoestrogenism, characterized by menstrual cycle disturbances with irregular and prolonged intervals, eventually leading to secondary amenorrhea. It may also present as primary amenorrhea when ovarian function is lost before the onset of puberty. Laboratorially, it is characterized as a state of hypergonadotropic hypogonadism. Its prevalence is approximately 3.5 %, and reported etiologies include genetic, autoimmune, and iatrogenic factors such as ovarian surgeries, chemotherapy and radiotherapy, as well as metabolic disorders and infections. However, in a lot of cases, the cause of POI often remains unidentified and is described as idiopathic. Women with POI have unique needs that must be individually addressed.^1^, ^2^, ^3^

Synonyms of POI include primary ovarian insufficiency, premature ovarian failure, early ovarian failure, premature ovarian dysfunction, and hypergonadotropic amenorrhea. It is important to highlight that “premature ovarian insufficiency” is the term recommended by the European Society of Human Reproduction and Embryology (ESHRE) and the Brazilian Federation of Gynecology and Obstetrics Associations (FEBRASGO).^1^^,^^2^ This terminology highlights the importance of managing this condition differently from a physiological menopause, since there are particular characteristics that must be considered at the time of diagnosis and in prescribing appropriate treatment.

**Fig. 1:**
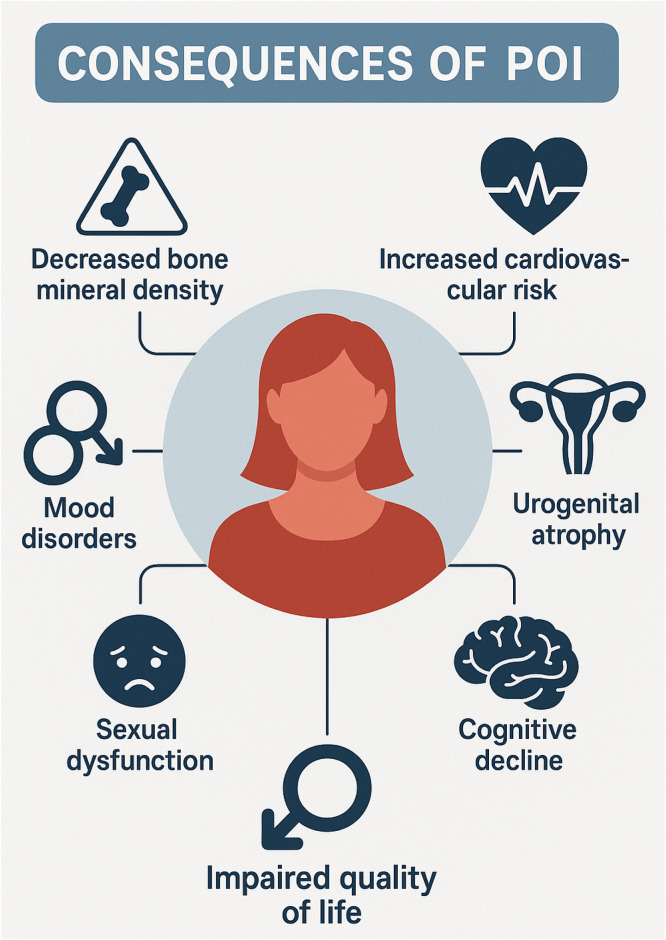
Consequences of POI without treatment.

Beyond the impact on fertility, premature ovarian insufficiency affects women during the prime of their productive lives ‒ typically young, sexually active individuals who have not yet reached the expected age of natural menopause. This condition imposes not only clinical challenges but also significant emotional and social burdens, requiring care strategies that go beyond the conventional approach used in menopause management. Hormone Therapy (HT) is considered mandatory and should be initiated early to restore adequate estrogen levels and prevent the multiple consequences of hypoestrogenism, including sexual dysfunction, bone mass loss, mood disorders, vasomotor symptoms, and increased cardiovascular risk^1^, ^2^, ^3^, ^4^, ^5^, ^6^ (Fig. 1).

Unlike women in physiological menopause, those with POI require higher doses of estrogen, appropriate for the age at which ovarian failure occurred, to adequately support physical, sexual, and emotional well-being. Transdermal 17-β-estradiol or oral administration at full doses is preferred, and the therapeutic regimen must be individualized based on the presence of a uterus and the need for progestogens to protect the endometrium.^6^ Furthermore, it is essential to recognize that many of these women may live for decades in a state of hypoestrogenism if inadequately treated, making hormone replacement therapy a central component of care, with positive effects on quality of life, cognitive function, and longevity.^1^, ^2^, ^3^, ^4^, ^5^, ^6^

This review aims to synthesize recent data and recommendations regarding the diagnosis and management of POI, emphasizing hormonal treatment strategies and long-term follow-up.

## Methods

This is a narrative review based on the official FEBRASGO protocol for POI (2021) and the updated international guideline published by the European Society of Human Reproduction and Embryology (ESHRE) in 2024. Additionally, a literature search was conducted in PubMed and Scopus using terms such as “premature ovarian insufficiency”, “hormone therapy”, and “fertility preservation”, including articles published in English or Portuguese in the last 10-years.

## Results and discussion

### Etiology and diagnosis

POI may arise from genetic abnormalities (e.g., Turner syndrome, FMR1 premutation), autoimmune diseases (e.g., thyroiditis), iatrogenic causes (e.g., chemotherapy), or environmental exposures. However, in many cases, the cause remains idiopathic (Table 1). Diagnosis requires the presence of amenorrhea for ≥ 4-months and elevated FSH levels (> 25 mIU/mL) on two occasions spaced at least four weeks apart.^1^, ^2^, ^3^, ^4^, ^5^

**Table 1:** Etiology and diagnostic criteria for Premature Ovarian Insufficiency (POI).

Aspect	Details
Etiology	• Genetic: Turner syndrome, FMR1 premutation
	• Autoimmune: Autoimmune thyroiditis, Addison's disease
	• Iatrogenic: Chemotherapy, radiotherapy, oophorectomy
	• Environmental: Toxins, infections
	• Idiopathic: Many cases have no identifiable cause
Diagnostic Criteria	FSH > 25 mIU/mL on two occasions at least 4-weeks apart
Recommended Workup	• TSH serum levels
	• Serum FSH levels (2×, ≥ 4-weeks apart)
	• Karyotype (especially in primary amenorrhea)
	• FMR1 premutation screening (in familial cases)
Not Recommended	Anti-Müllerian Hormone (AMH) is not recommended as a substitute for FSH in the diagnosis of POI
	Anti-TPO is not recommended in the diagnosis of POI

Additional evaluations include thyroid function tests, karyotype (especially in cases of primary amenorrhea), and screening for the FMR1 premutation in familial cases. The utility of AMH as a diagnostic tool is limited and not recommended as a substitute for FSH.^1^

Although the request for 21-OH-hydroxylase is suggested in the literature, it may have limited use in non-specialized centers. Anti-TPO is no longer recommended given its high prevalence in the general population.^1^

So, clinicians should consider investigating POI in any woman under the age of 40 who presents with prolonged menstrual cycles, infrequent menstruation, or amenorrhea lasting four months or more. A thorough clinical history should guide the selection of complementary tests, especially in identifying potential autoimmune, genetic, or iatrogenic causes. The first-line investigation includes serum FSH measurement, repeated at least four weeks apart, alongside estradiol levels. In special cases of primary amenorrhea or strong family history, karyotype analysis and FMR1 premutation testing are indicated. However, these tests can be indicated for all non-iatrogenic POIs. The presence of a Y chromosome or a fragment of the Y chromosome in the karyotype changes the approach, with gonadectomy being indicated due to the risk of malignancy. Thyroid function (TSH) should be assessed in all cases at diagnosis. Early recognition and timely investigation of POI are essential to prevent long-term complications and initiate appropriate hormonal therapy without delay.^6^, ^7^, ^8^, ^9^, ^10^, ^11^

### Clinical implications

POI is associated with significant morbidity, including decreased bone mineral density, increased cardiovascular risk, urogenital atrophy, mood disorders, and cognitive decline.^5^, ^6^, ^7^, ^8^, ^9^ Sexual dysfunction and impaired quality of life are frequently reported, particularly in women not receiving hormone therapy.^11^^,^^12^

### Hormone therapy (HT)

HT is the mainstay of POI treatment and should be initiated as early as possible after diagnosis, in the absence of contraindications, and continued until the average age of natural menopause (∼50-years)^13^^,^^14^ (Table 2).

• **Estrogen therapy:** Transdermal 17β-estradiol (75–100 μg/day) is preferred due to its favorable metabolic profile. Oral formulations (estradiol valerate 2–4 mg/day) are also effective. In adolescents without the complete development of secondary sexual characteristics and still in the growth phase, estrogen should be initiated at low doses and titrated gradually to adult levels.^15^
• **Progestogens:** Required for endometrial protection in women with a uterus. Options include micronized progesterone, medroxyprogesterone acetate (Provera), norethindrone acetate, dihidrogesterone, or levonorgestrel-releasing intrauterine systems. Both cyclic and continuous regimens are acceptable.^16^
• **Contraceptive pills:** Combined oral contraceptives with 30 μg of ethinylestradiol may be used when contraception is desired, in a continuous regime without a hormone-free interval, but are not suitable for pubertal induction.^17^^,^^18^

**Table 2:** Recommendations of hormone therapy for POI.

Age Range	Medications and Comments
12–13 years	If no secondary sexual characteristics and elevated FSH: initiate low-dose estrogen.
	Estradiol (E2): • Transdermal: 12.5 μg/day (patch) • Oral micronized E2: 0.5 mg/day
12.5–15 years	Gradually increase the estrogen dose every 6-months until reaching adult doses.
	Estradiol (E2): • Transdermal: 25, 50, 100 μg/day (adult dose: 75–100 μg/day) • Oral E2: 0.5, 1.0, 1.5, 2.0 mg/day (adult dose: 2–4 mg/day)
14–16 years	Initiate progestogen after 2-years or upon first withdrawal bleeding (whichever occurs first), or based on ultrasound showing endometrial response.
	Progestogen for 14-days/month: • Oral micronized progesterone: 200 mg/day • Dydrogesterone: 5–10 mg/day • medroxyprogesterone acetate (Provera): 10 mg/day
16–50 years	Full-dose estrogen and progestogen therapy.
	Estradiol (E2): • Oral: 2–4 mg/day • Transdermal patch: 75–100 μg/day • Transdermal gel (sachet): 1.5–2 mg/day • Transdermal gel (0.75 mg/pump): 3‒4 dose/day; Transdermal spray (1.53 mg/spray): 3‒4 dose/day
	Progestogen: • Sequential regimen (14 consecutive days/month): • Oral micronized progesterone: 200–400 mg/day • Dydrogesterone: 10–20 mg/day • medroxyprogesterone acetate (Provera): 10 mg/day • Norethisterone: 10 mg/day
	• Continuous regimen (daily use): • Oral micronized progesterone: 200–400 mg/day • Dydrogesterone: 10–20 mg/day • medroxyprogesterone acetate (Provera): 5 mg/day • Norethisterone: 5 mg/day • LNG-IUD (52 mg)
	• Continuous combined oral contraceptive with 30 μg ethinylestradiol (especially when pregnancy risk exists and is not acceptable)
Above 50 years	Hormone therapy should follow postmenopausal guidelines and individual considerations

Source: ESHRE, European Society of Human Reproduction and Embryology; ASRM, American Society for Reproductive Medicine; IMS, International Menopause Society; CRE WHiRL, Centre for Research Excellence in Women’s Health in Reproductive Life.

Premature Ovarian Insufficiency: International Guideline 2024. ESHRE; 2024; Bondy CA; Turner Syndrome Study Group. Care of girls and women with Turner syndrome: a guideline of the Turner Syndrome Study Group. J Clin Endocrinol Metab. 2007;92(1):10–25; Furness S, Roberts H, Marjoribanks J, Lethaby A. Hormone therapy in postmenopausal women and risk of endometrial hyperplasia. Cochrane Database Syst Rev. 2012;8(8):CD000402; Gazarra LB, Bonacordi CL, Yela DA, Benetti-Pinto CL. Bone mass in women with premature ovarian insufficiency: a comparative study between hormone therapy and combined oral contraceptives. Menopause. 2020;27(10):1110–1116.

HT improves vasomotor symptoms, prevents bone loss, and may mitigate cardiovascular and cognitive risks. While some women with POI have reduced testosterone levels, routine androgen supplementation is not recommended unless sexual dysfunction is diagnosed and persists despite adequate estrogen replacement.^1^ Hormone therapy should be supplemented according to the repercussions and symptoms presented. Thus, women who continue to have genitourinary complaints may receive supplementation with estrogen, lubricant, or vaginal moisturizer; in the presence of bone loss, calcium and vitamin D supplementation may be indicated.

### Androgenic therapy

Androgen replacement may be considered in women with Premature Ovarian Insufficiency (POI) who present with hypoactive sexual desire, provided that other potential causes have been properly excluded. Transdermal administration is the preferred route, as it ensures stable hormone levels with a lower risk of systemic adverse effects, and clinical monitoring is essential to assess both efficacy and safety. The most recent international guideline from ESHRE (2024) acknowledges androgen therapy as an optional adjunct in selected cases, complementing standard hormonal replacement therapy.^1^ A systematic review and meta-analysis found that women with POI exhibit significantly reduced serum levels of androgens, including testosterone and androstenedione, supporting the physiological rationale for considering androgen supplementation. However, available studies have not demonstrated improvements in bone mineral density with the addition of testosterone to conventional hormone therapy, and there are no robust data on fracture risk reduction or head-to-head comparisons of different androgen formulations.^19^ Further research is needed to clarify the long-term efficacy and safety of androgen therapy in this population.

When androgen replacement is considered, transdermal formulations are preferred due to their favorable pharmacokinetic profile and safety. In clinical practice, testosterone can be prescribed in a compounded transdermal gel using a Pentravan® base, at a dose ranging from 1 to 5 mg per day, depending on clinical response and tolerability. This dosing aims to restore serum testosterone levels to the premenopausal physiological range observed in women. The treatment should be individualized and initiated at the lowest effective dose, with gradual adjustments if necessary. Regular clinical follow-up is essential to evaluate symptom improvement, assess for potential adverse effects such as acne, hirsutism, or mood changes, and ensure that serum testosterone concentrations remain within the female reference range. Testosterone therapy in women should only be prescribed by healthcare professionals experienced in hormonal management and using well-established protocols for safety monitoring. Importantly, if symptoms of hypoactive sexual desire do not improve after adequate testosterone therapy, it is likely that other contributing factors ‒ such as psychological, relational, or contextual issues ‒ are involved and should be investigated and addressed through a multidisciplinary approach.^1^

### Fertility and preservation options

Although spontaneous pregnancy is rare (< 5 %), ovulation may occasionally resume. Women desiring pregnancy should be counseled about assisted reproduction techniques, especially oocyte donation.^20^ In situations of POI after cancer treatments, after infections, and due to autoimmune diseases, there is a greater possibility of transient ovarian insufficiency, with possible spontaneous ovulation. In women who do not wish to become pregnant, it is important to discuss contraception, always preferring methods that have the ability to preserve the consequences of hypoestrogenism, with emphasis on combined oral contraceptives containing 30 mcg of ethinylestradiol and levonorgestrel intrauterine system associated with oral or transdermal estrogen therapy.^1^

Fertility preservation is crucial in at-risk populations (e.g., cancer patients). Oocyte cryopreservation is recommended for post-pubertal patients, while ovarian tissue cryopreservation is an option for prepubertal girls or when immediate gonadotoxic therapy is needed.^20^, ^21^, ^22^

### Long-term monitoring

Patients with POI require regular assessment of cardiovascular risk, bone mineral density, thyroid function, and psychological well-being. DEXA scans are indicated at diagnosis and periodically thereafter to monitor bone mineral density, especially in women who are not on adequate hormone replacement therapy or have other risk factors for osteoporosis.^23^ In addition, serial evaluations of metabolic parameters are recommended. Fasting lipid profile (total cholesterol, LDL-C, HDL-C, and triglycerides) and fasting glucose or HbA1c should be measured at baseline and at regular intervals ‒ at least annually or more frequently in those with abnormal results or additional risk factors.

Anthropometric data should be monitored during follow-up, including body weight, Body Mass Index (BMI), waist circumference, and hip circumference, allowing for the calculation of Waist-to-Hip Ratio (WHR), a marker of central adiposity associated with cardiometabolic risk. Lifestyle evaluation should also be part of routine care, including assessment of physical activity patterns, dietary habits, smoking status, alcohol consumption, and sleep quality. Patients should receive guidance on adopting a healthy lifestyle, including regular exercise (preferably combining aerobic and resistance training), a balanced diet rich in calcium and vitamin D, smoking cessation, and stress management strategies.^1^

Psychosocial support, including screening for anxiety and depression, should be offered periodically, as POI can have significant emotional and quality-of-life impacts. Education and counseling are vital to enhance treatment adherence, ensure patient engagement, and improve long-term outcomes. Coordination with a multidisciplinary team ‒ including endocrinologists, gynecologists, dietitians, psychologists, and physical therapists ‒ can optimize the care of women with POI throughout their lifespan. Education and counseling are vital to enhance treatment adherence and improve long-term outcomes.

## Conclusion

POI is a chronic condition with extensive systemic and psychological consequences. Hormonal replacement therapy remains the cornerstone of treatment and must be tailored to the patient’s age, symptoms, reproductive desires, and comorbidities. Early diagnosis and a multidisciplinary approach are essential to preserve quality of life and prevent long-term complications.

## Declaration of competing interest

The authors declare no conflicts of interest.
